# Supplementary material for: Factors associated with maternal health services utilization in Pakistan: Evidence from Pakistan maternal mortality survey, 2019
Source: PLoS One. 2023 Nov 16;18(11):e0294225. doi: 10.1371/journal.pone.0294225 (PMC10653445; doi:10.1371/journal.pone.0294225)
Supplement: Attachment — Submitted filename: 0.PONE-D-22-31027_R2_reviewer.pdf [file pone.0294225.s005.pdf]

PLOS ONE

Factors associated with maternal health services utilization in Pakistan: evidence from Pakistan Maternal Mortality Survey, 2019

--Manuscript Draft--

**Manuscript Number:**	PONE-D-22-31027R2
**Article Type:**	Research Article
**Full Title:**	Factors associated with maternal health services utilization in Pakistan: evidence from Pakistan Maternal Mortality Survey, 2019
**Short Title:**	Factors associated with maternal health services utilization in Pakistan
**Corresponding Author:**	Raheel Shahab Khan, MS<br>Health Services Academy<br>Islamabad, PAKISTAN
**Keywords:**	antenatal care; skilled birth attendance; Pakistan; Demographic and Health Survey
**Abstract:**	Background<br><br>This study investigates the factors associated with maternal health services utilization in Pakistan using two indicators, ideal antenatal care (IANC) and skilled birth attendance (SBA).<br><br>Methods<br><br>This study used the Pakistan Maternal Mortality Survey 2019 data. We used binary logistic regression models to investigate the adjusted association between the outcome variables, separately for IANC and SBA, and the independent variables, education, wealth, parity and residence.<br><br>Results<br><br>Wealth showed a positive association with utilization of IANC (adjusted odds ratio [AOR]= 11.48, 95% CI = 7.76, 16.99) and SBA (AOR = 4.37, 95% CI = 3.30,5. 80). Age at birth was associated only with IANC use for women aged 35 or more years (AOR = 1.31, 95% CI = 1.06,1.62). Increase in odds ratio for utilization of IANC and SBA services were also observed for women with education. Women who had 3—5 child births had higher odds of using IANC and SBA than women had 1—2 or more than five births. Urban residency didn't show a significant association with IANC and SBA.<br><br>Conclusion<br><br>Women in the lower wealth quintile and women with no education had low utilization of ANC and SBA services compared to wealthy and educated women. A multipronged approach from the health and education sector could improve the utilization of maternal health services.
**Order of Authors:**	Farid Midhet, PhD<br>Mubashir Hanif, PhD<br>Raheel Shahab Khan, MS<br>Ikhlaq Ahmad, PhD<br>Samina Naeem Khalid, PhD<br>Shahzad Ali Khan, PhD
**Response to Reviewers:**	REVIEW COMMENTS TO THE AUTHOR<br><br>Reviewer #1: The authors have improved upon the original submission by addressing the discussion section. However, some important considerations remain and must be addressed:

Powered by Editorial Manager® and ProduXion Manager® from Aries Systems Corporation

Reviewer #1: Comment
1.They show that only around a fifth of the women avail IANC while 2/3 avail SBA. Statistically but more importantly contextually that makes these two very different phenomena. For example, age seems not to change SBA and there is no dose-dependance of education or parity suggesting (when there is a dramatic dose effect of over 6 children elsewhere), demand issues. It would useful for the authors to describe what is similar and different, in terms of which women avail which service, location, demographic and the channel of service delivery.
Answer: 1
There are 2/3 of women who have availed SBA services, but it does not mean that they had an Ideal ANC (IANC). We have explained that the trends of increasing use of ANC and SBA are parallel. However, the quality of ANC received by women is in question, which we wish to highlight. Ideal ANC means that they have the ANC visits & the required investigation in addition to the utilization of SBA services, the standards as explained below:
•four or more ANC visits to a skilled healthcare professional during pregnancy, with at least one visit in the first trimester;
•laboratory tests of blood and urine carried out at least once;
•blood pressure measure on each visit;
•prescription of iron tablets;
•counselling on nutrition during pregnancy; and
•at least one dose of tetanus toxoid vaccination. SBA was defined as the delivery conducted by a skilled birth attendant including doctor, nurse, midwife, community midwife, or Lady Health Visitor (LHV).
We argue that the quality of ANC is more important than simply visiting the health facility during pregnancy without being properly checked for potential risk factors, receiving iron-folic acid supplementation, getting anti-tetanus vaccination, and being informed about nutrition.
It would useful for the authors to describe what is similar and different, in terms of which women avail which service, location, demographic and the channel of service delivery.
The women who use SBA services are mostly affected by wealth quintile and education. The discussion points is that women in Baluchistan, Sindh and GB have relatively lower SBA services because of the longer distances to health facilities although other factors like parity and education are also in play.
Reviewer #1: Comment

2.A recommendation is made for reaching poor and rural women. However, their results show no difference in utilization by location. Further, given that over half of Pakistan's urban population lives in an urban slum (which would not be identified in this survey, it is likely that their being underserved is not being highlight (given the wealth and education associations that are described). This would suggest that the recommendation should be to focus predominantly on urban poor
Answer: 2
We have pointed out that both poverty and residence in rural areas are independent risk factors, and should be addressed. This impact is independent of the province of residence. There is definitely marked difference in rural and urban women.
a)The table 1 shows that urban women utilize IANC (27.9%) and rural women use (15.5) which shows significant difference.
b)Our study suggests strong relationship with lowest wealth quintile (only 3% IANC)
c)Yes, definitely urban poor should be the focus in a province, but since the data of PMMS was not segregated in the urban slums therefore this can be a limitation of the present study.
Reviewer #1: Comment

3.There is some discussion of supply side interventions. It is unclear given the correlates for low IANC that are described in the regression would mean much more utilization of better trained or equipped providers/ facilities would lead to higher uptake. It seems from the results presented that the problem is a lack of demand and recommendations may address that.
Answer: 3
We respectfully disagree and say that this is not the impression coming out of our results. We are focusing on quality of ANC rather than simply the supply of it; hence, it is a supply-side issue. If we will stress on IANC services, that will automatically include

	SBA services.<br>Additionally, if the pregnant women reach the health facilities and interact with the health system including a contact with the SBAs, this would increase their likelihood of opting for institutional delivery rather than home delivery by an untrained birth attendant.<br>Reviewer #1: Comment<br>4.A similar argument may be made for SBA. Pakistan saw rapid rise in uptake of SBA between 2012 and 2017, that was attributed by many to supply side interventions such as better equipped and 24/7 facilities. That advantage has been realized. Perhaps a more pronounced discussion of demand creation for both IANC and SBA, given demographic features of low utilizers may be proposed.<br>Answer: 4<br>Our focus is quality of ANC. However, we have mentioned interventions like Sehat Sahulat Program in the discussion and conclusion.<br>Reviewer #1: Comment<br>5.The discussion is still a bit long, repetitive (both of results and arguments) and circular (argument order need to be revisited for logical flow). It can be cut by at least<br>Answer: 5<br>We emphasize the importance of giving attention to the association between each factor and ANC.<br>Reviewer #1: Comment<br>6.I am not sure I agree with supply side recommendations that are presented, given the above points. These must be revisited to include a more sophisticated inclusion of context and demand issues<br>Answer: 6<br>Definitely, the supply side recommendation is needed, which we have highlighted that through LHWs and other community-based workers efforts should be made to improve knowledge of women about the importance of ANC services. There should also stress on the importance of blood tests and other necessary services.<br><br>Reviewer #2: Authors have made revisions that are acceptable and changes made are fine for the manuscript to be accepted.
**Additional Information:**	
**Question**	**Response**
**Financial Disclosure**<br><br>Enter a financial disclosure statement that describes the sources of funding for the work included in this submission. Review the submission guidelines for detailed requirements. View published research articles from *PLOS ONE* for specific examples.<br><br>This statement is required for submission and **will appear in the published article** if the submission is accepted. Please make sure it is accurate.	The authors received no specific funding for this work.

**Unfunded studies**<br>Enter: *The author(s) received no specific funding for this work.*<br><br>**Funded studies**<br>Enter a statement with the following details:<br>• Initials of the authors who received each award<br>• Grant numbers awarded to each author<br>• The full name of each funder<br>• URL of each funder website<br>• Did the sponsors or funders play any role in the study design, data collection and analysis, decision to publish, or preparation of the manuscript?<br>• **NO** - Include this sentence at the end of your statement: *The funders had no role in study design, data collection and analysis, decision to publish, or preparation of the manuscript.*<br>• **YES** - Specify the role(s) played.<br><br>* typeset	
**Competing Interests**<br><br>Use the instructions below to enter a competing interest statement for this submission. On behalf of all authors, disclose any competing interests that could be perceived to bias this work—acknowledging all financial support and any other relevant financial or non-financial competing interests.<br><br>This statement is required for submission and **will appear in the published article** if the submission is accepted. Please make sure it is accurate and that any funding sources listed in your Funding Information later in the submission form are also declared in your Financial Disclosure statement.<br><br>View published research articles from *PLOS ONE* for specific examples.	The authors have declared that no competing interests exist.

**NO authors have competing interests**<br><br>Enter: *The authors have declared that no competing interests exist.*<br><br>**Authors with competing interests**<br><br>Enter competing interest details beginning with this statement:<br><br>*I have read the journal's policy and the authors of this manuscript have the following competing interests: [insert competing interests here]*<br><br>* typeset	
**Ethics Statement**<br><br>Enter an ethics statement for this submission. This statement is required if the study involved:<br><br>• Human participants<br>• Human specimens or tissue<br>• Vertebrate animals or cephalopods<br>• Vertebrate embryos or tissues<br>• Field research<br><br>Write "N/A" if the submission does not require an ethics statement.<br><br>General guidance is provided below. Consult the submission guidelines for detailed instructions. **Make sure that all information entered here is included in the Methods section of the manuscript.**	N/A

**Format for specific study types**<br><br>**Human Subject Research (involving human participants and/or tissue)**<br>• Give the name of the institutional review board or ethics committee that approved the study<br>• Include the approval number and/or a statement indicating approval of this research<br>• Indicate the form of consent obtained (written/oral) or the reason that consent was not obtained (e.g. the data were analyzed anonymously)<br><br>**Animal Research (involving vertebrate animals, embryos or tissues)**<br>• Provide the name of the Institutional Animal Care and Use Committee (IACUC) or other relevant ethics board that reviewed the study protocol, and indicate whether they approved this research or granted a formal waiver of ethical approval<br>• Include an approval number if one was obtained<br>• If the study involved *non-human primates*, add *additional details* about animal welfare and steps taken to ameliorate suffering<br>• If anesthesia, euthanasia, or any kind of animal sacrifice is part of the study, include briefly which substances and/or methods were applied<br><br>**Field Research**<br><br>Include the following details if this study involves the collection of plant, animal, or other materials from a natural setting:<br>• Field permit number<br>• Name of the institution or relevant body that granted permission	
**Data Availability**<br><br>Authors are required to make all data underlying the findings described fully available, without restriction, and from the time of publication. PLOS allows rare exceptions to address legal and ethical concerns. See the PLOS Data Policy and FAQ for detailed information.	Yes - all data are fully available without restriction

A Data Availability Statement describing where the data can be found is required at submission. Your answers to this question constitute the Data Availability Statement and **will be published in the article**, if accepted.<br><br>**Important:** Stating 'data available on request from the author' is not sufficient. If your data are only available upon request, select 'No' for the first question and explain your exceptional situation in the text box.<br><br>Do the authors confirm that all data underlying the findings described in their manuscript are fully available without restriction?	
**Describe where the data may be found in full sentences. If you are copying our sample text, replace any instances of XXX with the appropriate details.**<br><br>• If the data are **held or will be held in a public repository**, include URLs, accession numbers or DOIs. If this information will only be available after acceptance, indicate this by ticking the box below. For example: *All XXX files are available from the XXX database (accession number(s) XXX, XXX.*).<br>• If the data are all contained **within the manuscript and/or Supporting Information files**, enter the following: *All relevant data are within the manuscript and its Supporting Information files.*<br>• If neither of these applies but you are able to provide **details of access elsewhere**, with or without limitations, please do so. For example:<br><br>*Data cannot be shared publicly because of [XXX]. Data are available from the XXX Institutional Data Access / Ethics Committee (contact via XXX) for researchers who meet the criteria for access to confidential data.*<br><br>*The data underlying the results presented in the study are available from (include the name of the third party*	All relevant data are within the manuscript and its Supporting Information files.

and contact information or URL).<br>• This text is appropriate if the data are owned by a third party and authors do not have permission to share the data.<br><br>* typeset	
Additional data availability information:	

Manuscript Click here to access/download;Manuscript;Manuscript.docx

RESEARCH ARTICLE

Factors associated with maternal health services utilization in Pakistan: evidence from Pakistan Maternal Mortality Survey, 2019

Farid Midhet[1], Mubashir Hanif[1], Samina Naeem Khalid[1], Raheel Shahab Khan[1*], Ikhlaq Ahmad[1], Shahzad Ali Khan[1]

[1] Health Services Academy, Islamabad, Pakistan

[*] raheel@hsa.edu.pk (RSK)

Abstract

Background

This study investigates the factors associated with maternal health services utilization in Pakistan using two indicators, ideal antenatal care (IANC), defined as the pregnant woman receiving all the essential services included in standard antenatal care, and skilled birth attendance (SBA).

Methods

This study used the Pakistan Maternal Mortality Survey 2019 data. We used binary logistic regression models to investigate the adjusted association between the outcome variables, separately for IANC and SBA, and the independent variables, education, wealth, parity, and residence.

Results

Wealth showed a positive association with utilization of IANC (adjusted odds ratio [AOR]= 11.48, 95% CI = 7.76, 16.99) and SBA (AOR = 4.37, 95% CI = 3.30,5. 80). Age at birth was associated only with IANC use for women aged 35 or more years (AOR = 1.31, 95% CI = 1.06,1.62). Increase in odds ratio for utilization of IANC and SBA services were also observed for women with education. Women who had 3—5 previous live births had higher odds of using IANC and SBA than women who had 1—2 or more than five previous live births. Urban residency was not correlated with either IANC or SBA.

Conclusion

Women in the lower wealth quintile and women with no education had low utilization of ANC and SBA services compared to wealthy and educated women. A multipronged approach from the health and education sector could improve the utilization of maternal health services.

Introduction

Pakistan, a lower-middle income country, faces enormous challenges in improving on key maternal health indicators. Public spending on health as a percentage of Gross Domestic Product (GDP) is just 1.2%, making out of pocket (OOP) expenditure as the primary mode of healthcare finance [1]. Perinatal mortality rate is 57 deaths per 1,000 pregnancies [2]. Between 2006 and 2019, the maternal mortality ratio (MMR) declined from 276 maternal deaths per 100,000 live births to 186, a mere 33% decline with an average annual rate of reduction of 2.7% [3, 4].

There is a substantial gap between developed countries and developing countries in the maternal health indicators. According to global estimate of MMR for 2017, about 94% of the global maternal deaths occurred in lower and middle-income countries (LMICs). About 68% of the world's total maternal deaths occur in sub-Saharan Africa and another 19% in Southern Asia [5]. Within countries, this skewness of maternal deaths is primarily concentrated in the poor segment. An assessment of maternal mortality between 1990 and 2015 shows that the poorest women accounted for 80% of maternal deaths, up from 68% in 1990 [6]. Poorest women are less likely to access and afford maternal health services than the wealthiest [7, 8].

Maternal health services—which include antenatal care (ANC), skilled birth attendance (SBA), emergency obstetric and neonatal care (EmONC), post-natal care, and family planning—are essential interventions for reducing the risk of maternal and neonatal deaths, and long-lasting morbidity. Ensuring healthy lives of mothers is one of the targets of the Sustainable Development Goals that calls for reducing the global MMR to less than 70 maternal deaths per 100,000 live births by 2030 (SDG 3.1). This requires an average annual rate of reduction of 6.1% (from 2016—2030) globally—an ambitious target for LMICs [5].

The LMICs have made progress on improving maternal health indicators against their local targets that are specific to their context. In the period 2015—2021, use of SBA was 85%, up from 77% in 2008—2014 and about 20 percentage points from period around 2001 [9]; whereas the use of ANC (at least one visit) by 2016 was 86% [10]. However, the quality of these services lags the coverage and deviates greatly from the recommended checks and guidelines. A study based on the demographic and health surveys (DHS) and multiple indicator cluster surveys (MICs) done in LMICs shows that although ANC coverage was reported to be high, only half of the women had their blood pressure checked and urine and blood tested. A third of the women did not receive any of these services at any point during their pregnancy [10]. Studies also show that there are wide variations in the qualifications and competencies of health personnel providing ANC and SBA within and across countries [11].

ANC and SBA are effective interventions for reducing maternal mortality [6]. ANC helps in identification of detrimental conditions such as anemia and high blood pressure, educates women on healthy practices and nutrition during pregnancy, and family planning. ANC also facilitates timely referral to specialized health facility in case of complications during

pregnancy and childbirth. Several studies have shown that women using ANC are more likely to use SBA [12-14]. Although ANC is not a sufficient intervention as obstetric complications are usually unpredictable, it increases the probability of the woman delivering in a health facility where such complications can be identified and addressed.

Pakistan has observed modest increases in SBA and ANC use in recent years. About 69% deliveries were assisted by skilled birth attendants in 2017, up from 52% in 2012; whereas 86% of women received ANC from skilled health providers (doctor, nurse, midwife, lady health worker) in 2017, up from 73% in 2012 [2, 15].

This paper presents an assessment of pregnant women's access to maternal health services in Pakistan using two indicators, ANC and SBA, and their correlation with the woman's age at birth, wealth, education, parity, and residence.

Methods

Data

We used data from Pakistan Maternal Mortality Survey 2019 (PMMS 2019), publicly available from the Demographic and Health Surveys (DHS) program (dhsprogram.com). It is Pakistan's first exclusive national survey on maternal mortality. The survey was designed and conducted by the National Institute of Population Studies (NIPS) (https://nips.org.pk/) in collaboration with the DHS Program. The sample included 136,226 households in the four provinces (Balochistan, Sindh, Khyber Pakhtunkhwa, and Punjab), Azad Jammu and Kashmir (AJK), and Gilgit-Baltistan (GB). Births and deaths of last three years were recorded in each household, and 1,177 deaths of women in 15-49 years age-group were investigated using a verbal autopsy questionnaire to identify maternal deaths. Moreover, in a sub-sample of 10% households, 14,703 ever-married women aged 15-49 years were interviewed to identify complications of and health services utilization in pregnancy, delivery, and postpartum in the last three years.

Description of study variables

Our outcome variables were ideal ANC and SBA. There are concerns about counting a de facto ANC visit as a proper visit and debates on what and how many visits constitute a quality ANC service [16-18]. We defined 'ideal' ANC (IANC), opposed to a mere attendance of antenatal clinic, as the ANC that meets all of the following criteria: 1) four or more ANC visits to a skilled healthcare professional during pregnancy, with at least one visit in the first trimester; 2) laboratory tests of blood and urine carried out at least once; 3) blood pressure measure on each visit; 4) prescription of iron tablets; 5) counselling on nutrition during pregnancy; and 6) at least one dose of tetanus toxoid vaccination. SBA was defined as the delivery conducted by a skilled birth attendant including doctor, nurse, midwife, community midwife, or Lady Health Visitor (LHV).

We identified woman's age at birth (< 25 years, 25-34 years, $\geq$ 35 years), education (no schooling, less than 10th grade, 10th grade or higher), wealth quintiles, parity (1-2, 3-5, and $\geq$ 5 previous live births), and urban/rural residence as independent variables to separately estimate their effects on each of the two outcome variables. Both types of variables were created through recoding of the raw data available from the PMMS 2019 data files.

Statistical analysis

We generated cross-tabulation between the dependent and independent variables, estimating the significance of the relationship through unadjusted odds ratio and Chi-squared test. We used binary logistic regression models to investigate the adjusted association between the outcome variables (separately for IANC and SBA) and the independent variables (education, wealth, and residence), after adjusting for the effects of age, parity, and prior history of poor pregnancy outcomes. The binary logistic regression results generated adjusted odds ratios (exponentiation of beta coefficients) and their 95% confidence intervals. Diagnostic tests on binary logistic regression analyses were also carried out including the Hosmer-Lemeshow Goodness of Fit Test. For testing collinearity between independent variables, we used the raw data (where age, parity, education, and wealth index were continuous variables) and conducted linear regression analysis keeping parity as dependent variable and calculating the correlation matrix and variance inflation factor. All these tests were found to be within acceptable limits. The statistical significance (p-value) is also reported. We used SPSS (v.19) for estimating the results.

Results

Table 1 shows the use of IANC and SBA services by sociodemographic factors. The overall proportion of women who received IANC was only 20.5%. The descriptive results indicated that the proportion receiving IANC and SBA services was higher among women who in the age group 25-34 years, had 10th grade or higher education, were in the highest wealth quintile and had 1-2 children.

Table 1

Descriptive statistics on use of maternal health services, %

	IANC	SBA
**Age at birth**		
< 25 years	18.9	70.9
25- 34 years	22.5	70.7
$\geq$ 35 years	17.1	64.3
**Education**		
No schooling	7.5	58
< 10th grade	22.6	76.6
10th grade or higher	43.2	84.6
**Wealth quintile**		

Lowest	3	45.9
Q2	10	62
Middle	17.7	74
Q4	31.2	82.9
Highest	47.2	88.6
**Parity**		
1-2 births	27.3	76.2
3-5 births	19	69.1
$\geq$ 6 births	6.5	53.2
**Residence**		
Urban	27.9	78.1
Rural	15.1	63.2
**Region of residence**		
AJK	35.8	77.2
GB	18.9	61.3
Balochistan	3.5	53.1
KP	17.2	70.3
Sindh	16.8	67.3
Punjab	27.9	78.3

IANC, ideal antenatal care

SBA, skilled birth attendance

Urban women were nearly two times higher, 28%, to receive IANC than rural women (15%). It was found that women with 10th grade or higher education were about one and a half times more likely (85%) to receive assistance from SBA during birth than the uneducated mothers (58%).

Table 2 presents the regression results for association between IANC and SBA use and sociodemographic factors. Wealth quintile showed a significantly positive association with IANC and SBA use. The odds of using IANC services were 11.48 times [95% CI = 7.76, 16.99] more for women in the highest wealth quintile than for women in the lowest quintile. The odds of SBA use also increased with each increase in the wealth level.

Table 2

Associations of sociodemographic outcomes with IANC and SBA utilization

	IANC		SBA	
	AOR	conf. interval	AOR	conf. interval
**Age at birth**				
< 25 years (ref.)	-	-	-	-
25- 34 years	0.80	0.67 - 0.96	0.98	0.83 - 1.16
$\geq$ 35 years	1.31**	1.06 - 1.62	1.14	0.96 - 1.36
**Education**				
No schooling (ref.)	-	-	-	-

1- 9 grades	1.88**	1.53 - 2.31	1.32*	1.13 - 1.55
≥ 10th grade	3.24**	2.63 - 3.99	1.28*	1.04 - 1.56
**Wealth quintile**				
Lowest (ref.)	-	-	-	-
Second	2.75**	1.88–4.02	1.67**	1.41 - 1.97
Middle	4.18**	2.87–6.08	2.52**	2.08 - 3.05
Fourth	7.17**	4.91–10.48	3.45**	2.74 - 4.33
Highest	11.48**	7.76–16.99	4.37**	3.30 - 5. 80
**Parity**				
1-2 births (ref.)	-	-	-	-
3-5 births	2.71**	1.94 - 3.79	1.72**	1.38 - 2.14
≥ 6 births	1.81**	1.32 - 2.47	1.33**	1.11 - 1.60
**Residence**				
Rural (ref.)	-	-	-	-
Urban	0.95	0.82 – 1.11	1.10	0.95 - 1.26

* $p < 0.05$, ** $p < 0.001$

AOR, adjusted odds ratio

Adjusted variables include: mother's age at birth, wealth, education, parity and place of residence

IANC, ideal antenatal care

SBA, skilled birth attendance

Age at birth was significantly associated only with IANC use for women aged 35 or more years [Odds ratio = 1.31, 95% CI = 1.06, 1.62]. It, however, was not associated with SBA. Increase in odds ratio for utilization of IANC and SBA services were also observed for women with education. Women who had 3—5 child births had higher odds of using IANC and SBA than women had 1—2 or more than five births. Women who had 1—2 child births were less likely to use IANC and SBA. Place of residence didn't show a significant association with IANC and SBA.

Discussion

The existing evidence on the effect of sociodemographic factors on utilization of maternal health services in Pakistan is based on a *de facto* definition of ANC, which count a visit during pregnancy to a healthcare provider as ANC regardless of the quality of the visit. We used a comprehensive definition of ANC that includes the essential medical check-ups, laboratory tests, and communication with healthcare provider to reflect the completeness and quality of ANC. We also used a nationally representative sample from the first-ever exclusive survey on maternal mortality and morbidity in Pakistan.

The purpose of this study was to assess the determinants of maternal healthcare utilization represented by ANC and SBA. Several studies have explored the factors affecting ANC visits,

but there exists, to our knowledge, no study in Pakistan that uses *ideal* ANC. There is also little empirical evidence around the factors affecting SBA in Pakistan. We have shown that ANC utilization and use of SBA have strong association with mother's wealth, education, and parity.

According to the Pakistan Demographic and Health Surveys (PDHS), ANC visits have increased over the years. The proportion of women using ANC (for the most recent birth) was 61% in 2006-07, 73% in 2012-13, and 86% in 2017-18 [2]. However, only 51% of the women had four or more visits in 2017-18 as recommended by the WHO's 2002 focused ANC (FANC) model [2, 19]. The components of ANC, as computed in PDHS 2017-18, were not uniform across the visits for all women. Only 55% had ANC visit in the first trimester, 89% percent had their blood pressure checked, 70% had their urine and blood samples taken, 69% received tetanus toxoid vaccination, and 70% received counselling on balance diet [2]. Including these components in the *ideal* ANC, we observed in the PMMS 2019 data that only 20.5% women received proper ANC.

We found that wealth was the strongest predictor of ANC and SBA use. This underlines the high out of pocket expenditure in Pakistan that poses financial constraints for poor women seeking maternal health services [20]. Several studies within Pakistan and other developing countries support positive association between wealth and 'ANC and SBA' use [21-25]. This suggests inequity in service utilization imposed by wealth status and that removing the financial barriers to the poor can increase uptake of ANC and SBA. As Pakistan gains significant improvement in the coverage indicators of ANC and SBA, the questions about equity and quality of care remain. This could be one reason that Pakistan has not seen a corresponding decrease in maternal, perinatal, and neonatal mortality – having the highest levels of these indicators among all its neighbouring countries except Afghanistan. There is a need to reach out to the poor and the rural women who do not have access to modern medical care during pregnancy and childbirth. Moreover, we found that only about one fifth of the pregnant women received ANC that was appropriate and complete, and about 80% of the pregnant women who seek ANC are unable to receive the full benefits of ANC because of the poor quality of care in the health facilities. This should be a cause of concern for the policymakers.

Studies show that demand side financing (DSF) can improve utilization of maternal health services. For instance, the maternal health voucher programmes in Bangladesh and Kenya were associated with increase in facility-based deliveries [26, 27]. Similarly, a study on the effect of a voucher scheme in a rural district of Pakistan showed a positive association with facility-based deliveries [28]. At present, Pakistan has started demand-side healthcare financing programs on federal and provincial levels: Sehat Sahulat Program (federal), Sehat Card Plus (Khyber Pakhtunkhwa), Sehat Hifazat (Gilgit Baltistan). These are insurance programs—financed with general tax revenues—that provide free healthcare services, including facility-based delivery services, to beneficiaries [29, 30]. Access to maternity services—caesarean and normal delivery—is universal in Khyber Pakhtunkhwa, whereas the federal program is also planning to make it universal in Punjab and areas administered by the

federal government. As the programs have enrolled all the poorest population in the respective regions, it could be expected that the proportion of women using institutional deliveries increase among women in the poor wealth quintile. However, high risks areas in Sindh and Balochistan do not have such programs in place. Our results showed that the proportion of women receiving IANC and SBA was the lowest in Balochistan and Sindh. Our results showed that reducing the financial barriers to maternity services for women could improve uptake of ideal ANC and coverage of SBA.

Woman's age at birth may affect the utilization of maternal health services; however, the results may be context based. This study observed that the odds of using IANC by women of age 35 or more years were higher compared to women aged less than 25 years. This could be because of increased risks in women older than 35 years of age which make them cautious about their health and seek ANC, while the healthcare providers also give them more attention resulting into a greater proportion receiving ideal ANC. Similarly, our results showed that women with more than two childbirths were more likely to use ANC and SBA services as compared to women with their first-born or second-born child. This finding is in contrast with the general view by several studies that ANC and SBA utilization is higher for women with first-born child (primipara women) than multiparous women [25, 28, 31, 32]. According to our results the odds of using ANC and SBA are higher among women having three to five births than women having one or two births. One possible explanation for this is that women who have faced complications or unpleasant experience in their first birth are more likely to use ANC and SBA, and also of the healthcare providers giving them more attention in the form of ideal ANC [33].

We found a positive association between women education and utilization of ANC and SBA services, which is in line with numerous studies that highlights the positive effect of women education on utilization of maternal health services [13, 14, 21, 22, 33]. Generally, education broadens the understanding of women around health risks, warnings, and healthy practices during pregnancy. It also gives women more autonomy to make health related decisions for themselves [34]. Such autonomy may be reinforced by the financial status of women as educated women are likely to be in well-off families and/or work in a formal sector compared to uneducated women. The positive role of education highlights the importance of provision of school-based education to adolescent girls. According to the PMMS data, 52% of the women had no education and the proportion of women with no education in the lowest quintile was a staggering 91% [4]. There is a need to invest in girl's education for long-term health outcomes and close the information gap about maternal health among uneducated adult women through local media (radio, television), health promotion activities, and maternal and child health (MCH) programs.

We tested the presence of multicollinearity between the independent variables, particularly wealth and education and parity and woman's age at birth, which were within acceptable limits. Nonetheless, our results should be interpreted with caution. It may be noted, however,

that even though these variables are commonly interrelated, their independent effects on pregnancy outcomes have also been established.

This study has certain limitations: Since this was a household survey, the exact parameters of an Ideal ANC could not be captured. We did not use the WHO ANC model of eight ANC visits with SBA, (recommended to lower perinatal mortality and enhance the woman's experience with healthcare), as it would further limit the number of women receiving ideal ANC. Hence the ideal ANC definition adopted in this study may be regarded as the bare minimum that a pregnant woman should receive. We did not control for a poor pregnancy outcome in the past (pregnancy loss and neonatal death), which may play a role in increasing the uptake of maternal health services to avoid unpleasant experiences in future. We did not include other factors contributing the socioeconomic status of the household such as husband's education and occupation, because we used the wealth index and quintiles reported by the Survey, which reflects the overall socioeconomic status of the household. Finally, we have included in our model both the wealth quintiles and woman's education as independent variables determining the likelihood of ANC and SBA use; we believe that the woman's education is an important determinant of her empowerment and ability to use health services independent of the household's wealth quintile.

Conclusion

The most important message from this study is that only one-fifth of pregnant women in Pakistan receive comprehensive ANC, and that poorer women and those with low education are less likely to receive proper antenatal care and skilled birth attendance. It seems that, in spite of the significant increase in the proportion of pregnant women visiting the health facilities for ANC, the care provided to them is incomplete and of poor quality. The federal and provincial governments need to improve the quality of ANC through health systems strengthening, training and supervision of healthcare providers, and by expanding the network of health insurance for the poor. In this regard, Sehat Sahulat Program can be utilized which provides improved access and good quality medical services to the poor population through a health insurance scheme.

There is also a need to develop and enforce guidelines for healthcare providers, both in the public and private sectors, to encourage them to provide complete and high-quality ANC services to the women. Lady Health Workers and other community-based workers should educate pregnant women about the importance of a comprehensive ANC including lab tests, counselling, tetanus immunization, and iron supplementation. Our study only provides initial insights into this important and persistent problem of reproductive health services in Pakistan and further work should be done for this important cause.

Acknowledgements

We extend our thanks to National Institute of Population Studies (NIPS), ICF and the DHS Program for collecting and providing the data that made this study possible.

Authors' contributions

FM and MH conceived and designed the study.
FM conducted the statistical analysis.

MH and RSK wrote the first draft of the manuscript.
FM, SNK, IK, and SAK revised the manuscript.

Funding

The authors received no specific funding for this work.

Competing interests

None

Data sharing statement

This study used data that was collected by the Demographic and Health Surveys Program and is available from www.dhsprogram.com

References

1. Government of Pakistan. Pakistan Economic Survey 2020-21. Finance Division 2022.
2. National Institute of Population Studies - NIPS/Pakistan, ICF. Pakistan Demographic and Health Survey 2017-18. Islamabad, Pakistan: NIPS/Pakistan and ICF, 2019.
3. National Institute of Population Studies - NIPS/Pakistan, Macro International. Pakistan Demographic and Health Survey 2006-07. Islamabad, Pakistan: NIPS/Pakistan and Macro International, 2008.
4. National Institute of Population Studies-NIPS. Pakistan Maternal Mortality Survey. Islamabad/Pakistan: NIPS/ICF, 2020.
5. World Health Organization. Trends in maternal mortality 2000 to 2017: estimates by WHO, UNICEF, UNFPA, World Bank Group and the United Nations Population Division. 2019.
6. Kassebaum NJ, Barber RM, Bhutta ZA, Dandona L, Gething PW, Hay SI, et al. Global, regional, and national levels of maternal mortality, 1990–2015: a systematic analysis for the Global Burden of Disease Study 2015. The Lancet. 2016;388(10053):1775-812.
7. Zhang R, Li S, Li C, Zhao D, Guo L, Qu P, et al. Socioeconomic inequalities and determinants of maternal health services in Shaanxi Province, Western China. PLoS One. 2018;13(9):e0202129.
8. Yadav AK, Sahni B, Jena PK. Education, employment, economic status and empowerment: Implications for maternal health care services utilization in India. Journal of Public Affairs. 2021;21(3):e2259.
9. United Nations Statistics Division (UNSD). The Sustainable Development Goals Report 2022.
10. Arsenault C, Jordan K, Lee D, Dinsa G, Manzi F, Marchant T, et al. Equity in antenatal care quality: an analysis of 91 national household surveys. The Lancet Global Health. 2018;6(11):e1186-e95.
11. Hobbs AJ, Moller A-B, Kachikis A, Carvajal-Aguirre L, Say L, Chou D. Scoping review to identify and map the health personnel considered skilled birth attendants in low-and-middle income countries from 2000–2015. PLoS One. 2019;14(2):e0211576.
12. McNellan C, Dansereau E, Colombara D, Palmisano E, Wallace M, Johanns C, et al. Uptake of antenatal care, and its relationship with participation in health services and behaviors: an analysis of the poorest regions of four Mesoamerican countries. Annals of Global Health. 2017;83(1).
13. Bhowmik J, Biswas R, Woldegiorgis M. Antenatal care and skilled birth attendance in Bangladesh are influenced by female education and family affordability: BDHS 2014. Public health. 2019;170:113-21.
14. Nyongesa C, Xu X, Hall JJ, Macharia WM, Yego F, Hall B. Factors influencing choice of skilled birth attendance at ANC: evidence from the Kenya demographic health survey. BMC pregnancy and childbirth. 2018;18(1):1-6.
15. National Institute of Population Studies - NIPS/Pakistan, ICF International. Pakistan Demographic and Health Survey 2012-13. Islamabad, Pakistan: NIPS/Pakistan and ICF International, 2013.
16. Kuhnt J, Vollmer S. Antenatal care services and its implications for vital and health outcomes of children: evidence from 193 surveys in 69 low-income and middle-income countries. BMJ open. 2017;7(11):e017122.
17. Hodgins S, D'Agostino A. The quality–coverage gap in antenatal care: toward better measurement of effective coverage. Global Health: Science and Practice. 2014;2(2):173-81.
18. Dowswell T, Carroli G, Duley L, Gates S, Gülmezoglu AM, Khan-Neelofur D, et al. Alternative versus standard packages of antenatal care for low-risk pregnancy. Cochrane Database of Systematic Reviews. 2015;(7).
19. World Health Organization. WHO recommendations on antenatal care for a positive pregnancy experience: World Health Organization; 2016.
20. Pakistan Bureau of Statistics. National Health Accounts Pakistan 2017-18. Islamabad, Pakistan: 2021.

21. Noh J-W, Kim Y-m, Lee LJ, Akram N, Shahid F, Kwon YD, et al. Factors associated with the use of antenatal care in Sindh province, Pakistan: A population-based study. PloS one. 2019;14(4):e0213987.
22. Islam MM, Masud MS. Determinants of frequency and contents of antenatal care visits in Bangladesh: Assessing the extent of compliance with the WHO recommendations. PloS one. 2018;13(9):e0204752.
23. Gupta RK, Shora TN, Verma AK, Jan R. Knowledge regarding antenatal care services, its utilization, and delivery practices in mothers (aged 15-49 years) in a rural area of North India. Trop J Med Res. 2015;18(2):89-94.
24. Teshale AB, Alem AZ, Yeshaw Y, Kebede SA, Liyew AM, Tesema GA, et al. Exploring spatial variations and factors associated with skilled birth attendant delivery in Ethiopia: geographically weighted regression and multilevel analysis. BMC Public Health. 2020;20(1):1-19.
25. Olakunde BO, Adeyinka DA, Mavegam BO, Olakunde OA, Yahaya HB, Ajiboye OA, et al. Factors associated with skilled attendants at birth among married adolescent girls in Nigeria: evidence from the Multiple Indicator Cluster Survey, 2016/2017. International health. 2019;11(6):545-50.
26. Schmidt J-O, Ensor T, Hossain A, Khan S. Vouchers as demand side financing instruments for health care: a review of the Bangladesh maternal voucher scheme. Health policy. 2010;96(2):98-107.
27. Bellows B, Kyobutungi C, Mutua MK, Warren C, Ezeh A. Increase in facility-based deliveries associated with a maternal health voucher programme in informal settlements in Nairobi, Kenya. Health policy and planning. 2013;28(2):134-42.
28. Agha S. Changes in the proportion of facility-based deliveries and related maternal health services among the poor in rural Jhang, Pakistan: results from a demand-side financing intervention. International journal for equity in health. 2011;10(1):1-12.
29. Ayub A, Khan RS, Khan SA, Hussain H, Tabassum A, Shehzad JA, et al. Progress of Khyber Pakhtunkhwa (Pakistan) towards universal health coverage. Journal of Ayub Medical College Abbottabad. 2018;30(3):481-4.
30. Sehat Sahulat Program. Financial protection 2021 [cited 2022 October 3]. Available from: https://sehatinsafcard.com/financial.php.
31. Zhou D, Zhou Z, Yang C, Ji L, Ghose B, Tang S. Sociodemographic characteristics associated with the utilization of maternal health services in Cambodia. BMC Health Services Research. 2020;20(1):1-12.
32. Larsen A, Exavery A, Phillips JF, Tani K, Kanté AM. Predictors of health care seeking behavior during pregnancy, delivery, and the postnatal period in rural Tanzania. Maternal and child health journal. 2016;20(8):1726-34.
33. Adu J, Tenkorang E, Banchani E, Allison J, Mulay S. The effects of individual and community-level factors on maternal health outcomes in Ghana. PloS one. 2018;13(11):e0207942.
34. Thaddeus S, Maine D. Too far to walk: maternal mortality in context. Social science & medicine. 1994;38(8):1091-110.

Click here to access/download
Supporting Information - Compressed/ZIP File Archive
data.zip

RESEARCH ARTICLE

Factors associated with maternal health services utilization in Pakistan: evidence from Pakistan Maternal Mortality Survey, 2019

Farid Midhet[1], Mubashir Hanif[1], Samina Naeem Khalid[1], Raheel Shahab Khan[1*], Ikhlaq Ahmad[1], Shahzad Ali Khan[1]

[1] Health Services Academy, Islamabad, Pakistan

[*] raheel@hsa.edu.pk (RSK)

Abstract

Background

This study investigates the factors associated with maternal health services utilization in Pakistan using two indicators, ideal antenatal care (IANC), defined as the pregnant woman receiving all the essential services included in standard antenatal care, and skilled birth attendance (SBA).

Methods

This study used the Pakistan Maternal Mortality Survey 2019 data. We used binary logistic regression models to investigate the adjusted association between the outcome variables, separately for IANC and SBA, and the independent variables, education, wealth, parity, and residence.

Results

Wealth showed a positive association with utilization of IANC (adjusted odds ratio [AOR]= 11.48, 95% CI = 7.76, 16.99) and SBA (AOR = 4.37, 95% CI = 3.30,5. 80). Age at birth was associated only with IANC use for women aged 35 or more years (AOR = 1.31, 95% CI = 1.06,1.62). Increase in odds ratio for utilization of IANC and SBA services were also observed for women with education. Women who had 3—5 previous live births had higher odds of using IANC and SBA than women who had 1—2 or more than five previous live births. Urban residency was not correlated with either IANC or SBA.

Conclusion

Women in the lower wealth quintile and women with no education had low utilization of ANC and SBA services compared to wealthy and educated women. A multipronged approach from the health and education sector could improve the utilization of maternal health services.

Introduction

Pakistan, a lower-middle income country, faces enormous challenges in improving on key maternal health indicators. Public spending on health as a percentage of Gross Domestic Product (GDP) is just 1.2%, making out of pocket (OOP) expenditure as the primary mode of healthcare finance [1]. Perinatal mortality rate is 57 deaths per 1,000 pregnancies [2]. Between 2006 and 2019, the maternal mortality ratio (MMR) declined from 276 maternal deaths per 100,000 live births to 186, a mere 33% decline with an average annual rate of reduction of 2.7% [3, 4].

There is a substantial gap between developed countries and developing countries in the maternal health indicators. According to global estimate of MMR for 2017, about 94% of the global maternal deaths occurred in lower and middle-income countries (LMICs). About 68% of the world's total maternal deaths occur in sub-Saharan Africa and another 19% in Southern Asia [5]. Within countries, this skewness of maternal deaths is primarily concentrated in the poor segment. An assessment of maternal mortality between 1990 and 2015 shows that the poorest women accounted for 80% of maternal deaths, up from 68% in 1990 [6]. Poorest women are less likely to access and afford maternal health services than the wealthiest [7, 8].

Maternal health services—which include antenatal care (ANC), skilled birth attendance (SBA), emergency obstetric and neonatal care (EmONC), post-natal care, and family planning—are essential interventions for reducing the risk of maternal and neonatal deaths, and long-lasting morbidity. Ensuring healthy lives of mothers is one of the targets of the Sustainable Development Goals that calls for reducing the global MMR to less than 70 maternal deaths per 100,000 live births by 2030 (SDG 3.1). This requires an average annual rate of reduction of 6.1% (from 2016—2030) globally—an ambitious target for LMICs [5].

The LMICs have made progress on improving maternal health indicators against their local targets that are specific to their context. In the period 2015—2021, use of SBA was 85%, up from 77% in 2008—2014 and about 20 percentage points from period around 2001 [9]; whereas the use of ANC (at least one visit) by 2016 was 86% [10]. However, the quality of these services lags the coverage and deviates greatly from the recommended checks and guidelines. A study based on the demographic and health surveys (DHS) and multiple indicator cluster surveys (MICs) done in LMICs shows that although ANC coverage was reported to be high, only half of the women had their blood pressure checked and urine and blood tested. A third of the women did not receive any of these services at any point during their pregnancy [10]. Studies also show that there are wide variations in the qualifications and competencies of health personnel providing ANC and SBA within and across countries [11].

ANC and SBA are effective interventions for reducing maternal mortality [6]. ANC helps in identification of detrimental conditions such as anemia and high blood pressure, educates women on healthy practices and nutrition during pregnancy, and family planning. ANC also facilitates timely referral to specialized health facility in case of complications during

pregnancy and childbirth. Several studies have shown that women using ANC are more likely to use SBA [12-14]. Although ANC is not a sufficient intervention as obstetric complications are usually unpredictable, it increases the probability of the woman delivering in a health facility where such complications can be identified and addressed.

Pakistan has observed modest increases in SBA and ANC use in recent years. About 69% deliveries were assisted by skilled birth attendants in 2017, up from 52% in 2012; whereas 86% of women received ANC from skilled health providers (doctor, nurse, midwife, lady health worker) in 2017, up from 73% in 2012 [2, 15].

This paper presents an assessment of pregnant women's access to maternal health services in Pakistan using two indicators, ANC and SBA, and their correlation with the woman's age at birth, wealth, education, parity, and residence.

Methods

Data

We used data from Pakistan Maternal Mortality Survey 2019 (PMMS 2019), publicly available from the Demographic and Health Surveys (DHS) program (dhsprogram.com). It is Pakistan's first exclusive national survey on maternal mortality. The survey was designed and conducted by the National Institute of Population Studies (NIPS) (https://nips.org.pk/) in collaboration with the DHS Program. The sample included 136,226 households in the four provinces (Balochistan, Sindh, Khyber Pakhtunkhwa, and Punjab), Azad Jammu and Kashmir (AJK), and Gilgit-Baltistan (GB). Births and deaths of last three years were recorded in each household, and 1,177 deaths of women in 15-49 years age-group were investigated using a verbal autopsy questionnaire to identify maternal deaths. Moreover, in a sub-sample of 10% households, 14,703 ever-married women aged 15-49 years were interviewed to identify complications of and health services utilization in pregnancy, delivery, and postpartum in the last three years.

Description of study variables

Our outcome variables were ideal ANC and SBA. There are concerns about counting a de facto ANC visit as a proper visit and debates on what and how many visits constitute a quality ANC service [16-18]. We defined 'ideal' ANC (IANC), opposed to a mere attendance of antenatal clinic, as the ANC that meets all of the following criteria: 1) four or more ANC visits to a skilled healthcare professional during pregnancy, with at least one visit in the first trimester; 2) laboratory tests of blood and urine carried out at least once; 3) blood pressure measure on each visit; 4) prescription of iron tablets; 5) counselling on nutrition during pregnancy; and 6) at least one dose of tetanus toxoid vaccination. SBA was defined as the delivery conducted by a skilled birth attendant including doctor, nurse, midwife, community midwife, or Lady Health Visitor (LHV).

We identified woman's age at birth (< 25 years, 25-34 years, ≥ 35 years), education (no schooling, less than 10th grade, 10th grade or higher), wealth quintiles, parity (1-2, 3-5, and ≥ 5 previous live births), and urban/rural residence as independent variables to separately estimate their effects on each of the two outcome variables. Both types of variables were created through recoding of the raw data available from the PMMS 2019 data files.

Statistical analysis

We generated cross-tabulation between the dependent and independent variables, estimating the significance of the relationship through unadjusted odds ratio and Chi-squared test. We used binary logistic regression models to investigate the adjusted association between the outcome variables (separately for IANC and SBA) and the independent variables (education, wealth, and residence), after adjusting for the effects of age, parity, and prior history of poor pregnancy outcomes. The binary logistic regression results generated adjusted odds ratios (exponentiation of beta coefficients) and their 95% confidence intervals. Diagnostic tests on binary logistic regression analyses were also carried out including the Hosmer-Lemeshow Goodness of Fit Test. For testing collinearity between independent variables, we used the raw data (where age, parity, education, and wealth index were continuous variables) and conducted linear regression analysis keeping parity as dependent variable and calculating the correlation matrix and variance inflation factor. All these tests were found to be within acceptable limits. The statistical significance (p-value) is also reported. We used SPSS (v.19) for estimating the results.

Results

Table 1 shows the use of IANC and SBA services by sociodemographic factors. The overall proportion of women who received IANC was only 20.5%. The descriptive results indicated that the proportion receiving IANC and SBA services was higher among women who in the age group 25-34 years, had 10th grade or higher education, were in the highest wealth quintile and had 1-2 children.

Table 1
Descriptive statistics on use of maternal health services, %

	IANC	SBA
**Age at birth**		
< 25 years	18.9	70.9
25- 34 years	22.5	70.7
≥ 35 years	17.1	64.3
**Education**		
No schooling	7.5	58
< 10th grade	22.6	76.6
10th grade or higher	43.2	84.6
**Wealth quintile**		

Lowest	3	45.9
Q2	10	62
Middle	17.7	74
Q4	31.2	82.9
Highest	47.2	88.6
**Parity**		
1-2 births	27.3	76.2
3-5 births	19	69.1
$\geq$ 6 births	6.5	53.2
**Residence**		
Urban	27.9	78.1
Rural	15.1	63.2
**Region of residence**		
AJK	35.8	77.2
GB	18.9	61.3
Balochistan	3.5	53.1
KP	17.2	70.3
Sindh	16.8	67.3
Punjab	27.9	78.3

IANC, ideal antenatal care

SBA, skilled birth attendance

Urban women were nearly two times higher, 28%, to receive IANC than rural women (15%). It was found that women with 10th grade or higher education were about one and a half times more likely (85%) to receive assistance from SBA during birth than the uneducated mothers (58%).

Table 2 presents the regression results for association between IANC and SBA use and sociodemographic factors. Wealth quintile showed a significantly positive association with IANC and SBA use. The odds of using IANC services were 11.48 times [95% CI = 7.76, 16.99] more for women in the highest wealth quintile than for women in the lowest quintile. The odds of SBA use also increased with each increase in the wealth level.

Table 2

Associations of sociodemographic outcomes with IANC and SBA utilization

	IANC		SBA	
	AOR	conf. interval	AOR	conf. interval
**Age at birth**				
< 25 years (ref.)	-	-	-	-
25- 34 years	0.80	0.67 - 0.96	0.98	0.83 - 1.16
$\geq$ 35 years	1.31**	1.06 - 1.62	1.14	0.96 - 1.36
**Education**				
No schooling (ref.)	-	-	-	-

1- 9 grades	1.88**	1.53 - 2.31	1.32*	1.13 - 1.55
≥ 10th grade	3.24**	2.63 - 3.99	1.28*	1.04 - 1.56
**Wealth quintile**				
Lowest (ref.)	-	-	-	-
Second	2.75**	1.88–4.02	1.67**	1.41 - 1.97
Middle	4.18**	2.87–6.08	2.52**	2.08 - 3.05
Fourth	7.17**	4.91–10.48	3.45**	2.74 - 4.33
Highest	11.48**	7.76–16.99	4.37**	3.30 - 5. 80
**Parity**				
1-2 births (ref.)	-	-	-	-
3-5 births	2.71**	1.94 - 3.79	1.72**	1.38 - 2.14
≥ 6 births	1.81**	1.32 - 2.47	1.33**	1.11 - 1.60
**Residence**				
Rural (ref.)	-	-	-	-
Urban	0.95	0.82 – 1.11	1.10	0.95 - 1.26

* $p < 0.05$, ** $p < 0.001$

AOR, adjusted odds ratio

Adjusted variables include: mother's age at birth, wealth, education, parity and place of residence

IANC, ideal antenatal care

SBA, skilled birth attendance

Age at birth was significantly associated only with IANC use for women aged 35 or more years [Odds ratio = 1.31, 95% CI = 1.06, 1.62]. It, however, was not associated with SBA. Increase in odds ratio for utilization of IANC and SBA services were also observed for women with education. Women who had 3—5 child births had higher odds of using IANC and SBA than women had 1—2 or more than five births. Women who had 1—2 child births were less likely to use IANC and SBA. Place of residence didn't show a significant association with IANC and SBA.

Discussion

The existing evidence on the effect of sociodemographic factors on utilization of maternal health services in Pakistan is based on a *de facto* definition of ANC, which count a visit during pregnancy to a healthcare provider as ANC regardless of the quality of the visit. We used a comprehensive definition of ANC that includes the essential medical check-ups, laboratory tests, and communication with healthcare provider to reflect the completeness and quality of ANC. We also used a nationally representative sample from the first-ever exclusive survey on maternal mortality and morbidity in Pakistan.

The purpose of this study was to assess the determinants of maternal healthcare utilization represented by ANC and SBA. Several studies have explored the factors affecting ANC visits,

but there exists, to our knowledge, no study in Pakistan that uses *ideal* ANC. There is also little empirical evidence around the factors affecting SBA in Pakistan. We have shown that ANC utilization and use of SBA have strong association with mother's wealth, education, and parity.

According to the Pakistan Demographic and Health Surveys (PDHS), ANC visits have increased over the years. The proportion of women using ANC (for the most recent birth) was 61% in 2006-07, 73% in 2012-13, and 86% in 2017-18 [2]. However, only 51% of the women had four or more visits in 2017-18 as recommended by the WHO's 2002 focused ANC (FANC) model [2, 19]. The components of ANC, as computed in PDHS 2017-18, were not uniform across the visits for all women. Only 55% had ANC visit in the first trimester, 89% percent had their blood pressure checked, 70% had their urine and blood samples taken, 69% received tetanus toxoid vaccination, and 70% received counselling on balance diet [2]. Including these components in the *ideal* ANC, we observed in the PMMS 2019 data that only 20.5% women received proper ANC.

We found that wealth was the strongest predictor of ANC and SBA use. This underlines the high out of pocket expenditure in Pakistan that poses financial constraints for poor women seeking maternal health services [20]. Several studies within Pakistan and other developing countries support positive association between wealth and 'ANC and SBA' use [21-25]. This suggests inequity in service utilization imposed by wealth status and that removing the financial barriers to the poor can increase uptake of ANC and SBA. As Pakistan gains significant improvement in the coverage indicators of ANC and SBA, the questions about equity and quality of care remain. This could be one reason that Pakistan has not seen a corresponding decrease in maternal, perinatal, and neonatal mortality – having the highest levels of these indicators among all its neighbouring countries except Afghanistan. There is a need to reach out to the poor and the rural women who do not have access to modern medical care during pregnancy and childbirth. Moreover, we found that only about one fifth of the pregnant women received ANC that was appropriate and complete, and about 80% of the pregnant women who seek ANC are unable to receive the full benefits of ANC because of the poor quality of care in the health facilities. This should be a cause of concern for the policymakers.

Studies show that demand side financing (DSF) can improve utilization of maternal health services. For instance, the maternal health voucher programmes in Bangladesh and Kenya were associated with increase in facility-based deliveries [26, 27]. Similarly, a study on the effect of a voucher scheme in a rural district of Pakistan showed a positive association with facility-based deliveries [28]. At present, Pakistan has started demand-side healthcare financing programs on federal and provincial levels: Sehat Sahulat Program (federal), Sehat Card Plus (Khyber Pakhtunkhwa), Sehat Hifazat (Gilgit Baltistan). These are insurance programs—financed with general tax revenues—that provide free healthcare services, including facility-based delivery services, to beneficiaries [29, 30]. Access to maternity services—caesarean and normal delivery—is universal in Khyber Pakhtunkhwa, whereas the federal program is also planning to make it universal in Punjab and areas administered by the

federal government. As the programs have enrolled all the poorest population in the respective regions, it could be expected that the proportion of women using institutional deliveries increase among women in the poor wealth quintile. However, high risks areas in Sindh and Balochistan do not have such programs in place. Our results showed that the proportion of women receiving IANC and SBA was the lowest in Balochistan and Sindh. Our results showed that reducing the financial barriers to maternity services for women could improve uptake of ideal ANC and coverage of SBA.

Woman's age at birth may affect the utilization of maternal health services; however, the results may be context based. This study observed that the odds of using IANC by women of age 35 or more years were higher compared to women aged less than 25 years. This could be because of increased risks in women older than 35 years of age which make them cautious about their health and seek ANC, while the healthcare providers also give them more attention resulting into a greater proportion receiving ideal ANC. Similarly, our results showed that women with more than two childbirths were more likely to use ANC and SBA services as compared to women with their first-born or second-born child. This finding is in contrast with the general view by several studies that ANC and SBA utilization is higher for women with first-born child (primipara women) than multiparous women [25, 28, 31, 32]. According to our results the odds of using ANC and SBA are higher among women having three to five births than women having one or two births. One possible explanation for this is that women who have faced complications or unpleasant experience in their first birth are more likely to use ANC and SBA, and also of the healthcare providers giving them more attention in the form of ideal ANC [33].

We found a positive association between women education and utilization of ANC and SBA services, which is in line with numerous studies that highlights the positive effect of women education on utilization of maternal health services [13, 14, 21, 22, 33]. Generally, education broadens the understanding of women around health risks, warnings, and healthy practices during pregnancy. It also gives women more autonomy to make health related decisions for themselves [34]. Such autonomy may be reinforced by the financial status of women as educated women are likely to be in well-off families and/or work in a formal sector compared to uneducated women. The positive role of education highlights the importance of provision of school-based education to adolescent girls. According to the PMMS data, 52% of the women had no education and the proportion of women with no education in the lowest quintile was a staggering 91% [4]. There is a need to invest in girl's education for long-term health outcomes and close the information gap about maternal health among uneducated adult women through local media (radio, television), health promotion activities, and maternal and child health (MCH) programs.

We tested the presence of multicollinearity between the independent variables, particularly wealth and education and parity and woman's age at birth, which were within acceptable limits. Nonetheless, our results should be interpreted with caution. It may be noted, however,

that even though these variables are commonly interrelated, their independent effects on pregnancy outcomes have also been established.

This study has certain limitations: Since this was a household survey, the exact parameters of an Ideal ANC could not be captured. We did not use the WHO ANC model of eight ANC visits with SBA, (recommended to lower perinatal mortality and enhance the woman's experience with healthcare), as it would further limit the number of women receiving ideal ANC. Hence the ideal ANC definition adopted in this study may be regarded as the bare minimum that a pregnant woman should receive. We did not control for a poor pregnancy outcome in the past (pregnancy loss and neonatal death), which may play a role in increasing the uptake of maternal health services to avoid unpleasant experiences in future. We did not include other factors contributing the socioeconomic status of the household such as husband's education and occupation, because we used the wealth index and quintiles reported by the Survey, which reflects the overall socioeconomic status of the household. Finally, we have included in our model both the wealth quintiles and woman's education as independent variables determining the likelihood of ANC and SBA use; we believe that the woman's education is an important determinant of her empowerment and ability to use health services independent of the household's wealth quintile.

Conclusion

The most important message from this study is that only one-fifth of pregnant women in Pakistan receive comprehensive ANC, and that poorer women and those with low education are less likely to receive proper antenatal care and skilled birth attendance. It seems that, in spite of the significant increase in the proportion of pregnant women visiting the health facilities for ANC, the care provided to them is incomplete and of poor quality. The federal and provincial governments need to improve the quality of ANC through health systems strengthening, training and supervision of healthcare providers, and by expanding the network of health insurance for the poor. In this regard, Sehat Sahulat Program can be utilized which provides improved access and good quality medical services to the poor population through a health insurance scheme.

There is also a need to develop and enforce guidelines for healthcare providers, both in the public and private sectors, to encourage them to provide complete and high-quality ANC services to the women. Lady Health Workers and other community-based workers should educate pregnant women about the importance of a comprehensive ANC including lab tests, counselling, tetanus immunization, and iron supplementation. Our study only provides initial insights into this important and persistent problem of reproductive health services in Pakistan and further work should be done for this important cause.

Acknowledgements

We extend our thanks to National Institute of Population Studies (NIPS), ICF and the DHS Program for collecting and providing the data that made this study possible.

Authors' contributions

FM and MH conceived and designed the study.
FM conducted the statistical analysis.

MH and RSK wrote the first draft of the manuscript.
FM, SNK, IK, and SAK revised the manuscript.

Funding
The authors received no specific funding for this work.

Competing interests
None

Data sharing statement
This study used data that was collected by the Demographic and Health Surveys Program and is available from www.dhsprogram.com

References

1. Government of Pakistan. Pakistan Economic Survey 2020-21. Finance Division 2022.
2. National Institute of Population Studies - NIPS/Pakistan, ICF. Pakistan Demographic and Health Survey 2017-18. Islamabad, Pakistan: NIPS/Pakistan and ICF, 2019.
3. National Institute of Population Studies - NIPS/Pakistan, Macro International. Pakistan Demographic and Health Survey 2006-07. Islamabad, Pakistan: NIPS/Pakistan and Macro International, 2008.
4. National Institute of Population Studies-NIPS. Pakistan Maternal Mortality Survey. Islamabad/Pakistan: NIPS/ICF, 2020.
5. World Health Organization. Trends in maternal mortality 2000 to 2017: estimates by WHO, UNICEF, UNFPA, World Bank Group and the United Nations Population Division. 2019.
6. Kassebaum NJ, Barber RM, Bhutta ZA, Dandona L, Gething PW, Hay SI, et al. Global, regional, and national levels of maternal mortality, 1990–2015: a systematic analysis for the Global Burden of Disease Study 2015. The Lancet. 2016;388(10053):1775-812.
7. Zhang R, Li S, Li C, Zhao D, Guo L, Qu P, et al. Socioeconomic inequalities and determinants of maternal health services in Shaanxi Province, Western China. PLoS One. 2018;13(9):e0202129.
8. Yadav AK, Sahni B, Jena PK. Education, employment, economic status and empowerment: Implications for maternal health care services utilization in India. Journal of Public Affairs. 2021;21(3):e2259.
9. United Nations Statistics Division (UNSD). The Sustainable Development Goals Report 2022.
10. Arsenault C, Jordan K, Lee D, Dinsa G, Manzi F, Marchant T, et al. Equity in antenatal care quality: an analysis of 91 national household surveys. The Lancet Global Health. 2018;6(11):e1186-e95.
11. Hobbs AJ, Moller A-B, Kachikis A, Carvajal-Aguirre L, Say L, Chou D. Scoping review to identify and map the health personnel considered skilled birth attendants in low-and-middle income countries from 2000–2015. PLoS One. 2019;14(2):e0211576.
12. McNellan C, Dansereau E, Colombara D, Palmisano E, Wallace M, Johanns C, et al. Uptake of antenatal care, and its relationship with participation in health services and behaviors: an analysis of the poorest regions of four Mesoamerican countries. Annals of Global Health. 2017;83(1).
13. Bhowmik J, Biswas R, Woldegiorgis M. Antenatal care and skilled birth attendance in Bangladesh are influenced by female education and family affordability: BDHS 2014. Public health. 2019;170:113-21.
14. Nyongesa C, Xu X, Hall JJ, Macharia WM, Yego F, Hall B. Factors influencing choice of skilled birth attendance at ANC: evidence from the Kenya demographic health survey. BMC pregnancy and childbirth. 2018;18(1):1-6.
15. National Institute of Population Studies - NIPS/Pakistan, ICF International. Pakistan Demographic and Health Survey 2012-13. Islamabad, Pakistan: NIPS/Pakistan and ICF International, 2013.
16. Kuhnt J, Vollmer S. Antenatal care services and its implications for vital and health outcomes of children: evidence from 193 surveys in 69 low-income and middle-income countries. BMJ open. 2017;7(11):e017122.
17. Hodgins S, D'Agostino A. The quality–coverage gap in antenatal care: toward better measurement of effective coverage. Global Health: Science and Practice. 2014;2(2):173-81.
18. Dowswell T, Carroli G, Duley L, Gates S, Gülmezoglu AM, Khan-Neelofur D, et al. Alternative versus standard packages of antenatal care for low-risk pregnancy. Cochrane Database of Systematic Reviews. 2015;(7).
19. World Health Organization. WHO recommendations on antenatal care for a positive pregnancy experience: World Health Organization; 2016.
20. Pakistan Bureau of Statistics. National Health Accounts Pakistan 2017-18. Islamabad, Pakistan: 2021.

21. Noh J-W, Kim Y-m, Lee LJ, Akram N, Shahid F, Kwon YD, et al. Factors associated with the use of antenatal care in Sindh province, Pakistan: A population-based study. PloS one. 2019;14(4):e0213987.
22. Islam MM, Masud MS. Determinants of frequency and contents of antenatal care visits in Bangladesh: Assessing the extent of compliance with the WHO recommendations. PloS one. 2018;13(9):e0204752.
23. Gupta RK, Shora TN, Verma AK, Jan R. Knowledge regarding antenatal care services, its utilization, and delivery practices in mothers (aged 15-49 years) in a rural area of North India. Trop J Med Res. 2015;18(2):89-94.
24. Teshale AB, Alem AZ, Yeshaw Y, Kebede SA, Liyew AM, Tesema GA, et al. Exploring spatial variations and factors associated with skilled birth attendant delivery in Ethiopia: geographically weighted regression and multilevel analysis. BMC Public Health. 2020;20(1):1-19.
25. Olakunde BO, Adeyinka DA, Mavegam BO, Olakunde OA, Yahaya HB, Ajiboye OA, et al. Factors associated with skilled attendants at birth among married adolescent girls in Nigeria: evidence from the Multiple Indicator Cluster Survey, 2016/2017. International health. 2019;11(6):545-50.
26. Schmidt J-O, Ensor T, Hossain A, Khan S. Vouchers as demand side financing instruments for health care: a review of the Bangladesh maternal voucher scheme. Health policy. 2010;96(2):98-107.
27. Bellows B, Kyobutungi C, Mutua MK, Warren C, Ezeh A. Increase in facility-based deliveries associated with a maternal health voucher programme in informal settlements in Nairobi, Kenya. Health policy and planning. 2013;28(2):134-42.
28. Agha S. Changes in the proportion of facility-based deliveries and related maternal health services among the poor in rural Jhang, Pakistan: results from a demand-side financing intervention. International journal for equity in health. 2011;10(1):1-12.
29. Ayub A, Khan RS, Khan SA, Hussain H, Tabassum A, Shehzad JA, et al. Progress of Khyber Pakhtunkhwa (Pakistan) towards universal health coverage. Journal of Ayub Medical College Abbottabad. 2018;30(3):481-4.
30. Sehat Sahulat Program. Financial protection 2021 [cited 2022 October 3]. Available from: https://sehatinsafcard.com/financial.php.
31. Zhou D, Zhou Z, Yang C, Ji L, Ghose B, Tang S. Sociodemographic characteristics associated with the utilization of maternal health services in Cambodia. BMC Health Services Research. 2020;20(1):1-12.
32. Larsen A, Exavery A, Phillips JF, Tani K, Kanté AM. Predictors of health care seeking behavior during pregnancy, delivery, and the postnatal period in rural Tanzania. Maternal and child health journal. 2016;20(8):1726-34.
33. Adu J, Tenkorang E, Banchani E, Allison J, Mulay S. The effects of individual and community-level factors on maternal health outcomes in Ghana. PloS one. 2018;13(11):e0207942.
34. Thaddeus S, Maine D. Too far to walk: maternal mortality in context. Social science & medicine. 1994;38(8):1091-110.

REVIEW COMMENTS TO THE AUTHOR

Please use the space provided to explain your answers to the questions above. You may also include additional comments for the author, including concerns about dual publication, research ethics, or publication ethics. (Please upload your review as an attachment if it exceeds 20,000 characters)

Reviewer #1: The authors have improved upon the original submission by addressing the discussion section. However, some important considerations remain and must be addressed:

Reviewer #1: Comment

1. **They show** that only around a fifth of the women avail IANC while 2/3 avail SBA. Statistically but more importantly contextually that makes these two very different phenomena. For example, age seems not to change SBA and there is no dose-dependance of education or parity suggesting (when there is a dramatic dose effect of over 6 children elsewhere), demand issues. It would useful for the authors to describe what is similar and different, in terms of which women avail which service, location, demographic and the channel of service delivery.

Answer: 1

There are 2/3 of women who have availed SBA services, but it does not mean that they had an Ideal ANC (IANC). We have explained that the trends of increasing use of ANC and SBA are parallel. However, the quality of ANC received by women is in question, which we wish to highlight. Ideal ANC means that they have the ANC visits & the required investigation in addition to the utilization of SBA services, the standards as explained below:

- four or more ANC visits to a skilled healthcare professional during pregnancy, with at least one visit in the first trimester;
- laboratory tests of blood and urine carried out at least once;
- blood pressure measure on each visit;
- prescription of iron tablets;
- counselling on nutrition during pregnancy; and
- at least one dose of tetanus toxoid vaccination. SBA was defined as the delivery conducted by a skilled birth attendant including doctor, nurse, midwife, community midwife, or Lady Health Visitor (LHV).

We argue that the quality of ANC is more important than simply visiting the health facility during pregnancy without being properly checked for potential risk factors, receiving iron-folic acid supplementation, getting anti-tetanus vaccination, and being informed about nutrition.

It would useful for the authors to describe what is similar and different, in terms of which women avail which service, location, demographic and the channel of service delivery.

The women who use SBA services are mostly affected by wealth quintile and education. The discussion points is that women in Baluchistan, Sindh and GB have relatively lower SBA services because of the longer distances to health facilities although other factors like parity and education are also in play.

Reviewer #1: Comment

2. **A recommendation** is made for reaching poor and rural women. However, their results show no difference in utilization by location. Further, given that over half of Pakistan's urban population lives in an urban slum (which would not be identified in this survey, it is likely that their being underserved is not being highlight (given the wealth and education associations that are described). This would suggest that the recommendation should be to focus predominantly on urban poor

Answer: 2

We have pointed out that both poverty and residence in rural areas are independent risk factors, and should be addressed. This impact is independent of the province of residence. There is definitely marked difference in rural and urban women.

a) The table 1 shows that urban women utilize IANC (27.9%) and rural women use (15.5) which shows significant difference.
b) Our study suggests strong relationship with lowest wealth quintile (only 3% IANC)
c) Yes, definitely urban poor should be the focus in a province, but since the data of PMMS was not segregated in the urban slums therefore this can be a limitation of the present study.

Reviewer #1: Comment

3. **There is some discussion** of supply side interventions. It is unclear given the correlates for low IANC that are described in the regression would mean much more utilization of better trained or equipped providers/ facilities would lead to higher uptake. It seems from the results presented that the problem is a lack of demand and recommendations may address that.

Answer: 3

We respectfully disagree and say that this is not the impression coming out of our results. We are focusing on quality of ANC rather than simply the supply of it; hence, it is a supply-side issue. If we will stress on IANC services, that will automatically include SBA services.

Additionally, if the pregnant women reach the health facilities and interact with the health system including a contact with the SBAs, this would increase their likelihood of opting for institutional delivery rather than home delivery by an untrained birth attendant.

Reviewer #1: Comment

4. **A similar argument** may be made for SBA. Pakistan saw rapid rise in uptake of SBA between 2012 and 2017, that was attributed by many to supply side interventions such as better equipped and 24/7 facilities. That advantage has been realized. Perhaps a more pronounced discussion of demand creation for both IANC and SBA, given demographic features of low utilizers may be proposed.

Answer: 4

Our focus is quality of ANC. However, we have mentioned interventions like Sehat Sahulat Program in the discussion and conclusion.

Reviewer #1: Comment

5. **The discussion** is still a bit long, repetitive (both of results and arguments) and circular (argument order need to be revisited for logical flow). It can be cut by at least

Answer: 5

We emphasize the importance of giving attention to the association between each factor and ANC.

Reviewer #1: Comment

6. **I am not sure** I agree with supply side recommendations that are presented, given the above points. These must be revisited to include a more sophisticated inclusion of context and demand issues

Answer: 6

Definitely the supply side recommendation is needed, which we have highlighted that through LHWs and other community-based workers efforts should be made to improve knowledge of women about the importance of ANC services. There should also stress on the importance of blood tests and other necessary services.

Reviewer #2: Authors have made revisions that are acceptable and changes made are fine for the manuscript to be accepted.
