# Supplementary material for: Factors associated with maternal health services utilization in Pakistan: Evidence from Pakistan maternal mortality survey, 2019
Source: PLoS One. 2023 Nov 16;18(11):e0294225. doi: 10.1371/journal.pone.0294225 (PMC10653445; doi:10.1371/journal.pone.0294225)
Supplement: Attachment — Submitted filename: Response to Reviewers.docx [file pone.0294225.s003.docx]

**Rebuttal Letter 1: Reviewer 1**

**1. Is the manuscript technically sound, and do the data support the conclusions?**

The manuscript must describe a technically sound piece of scientific research with data that supports the conclusions. Experiments must have been conducted rigorously, with appropriate controls, replication, and sample sizes. The conclusions must be drawn appropriately based on the data presented.

**Reviewer #1: Yes**

**2. Has the statistical analysis been performed appropriately and rigorously?**

**Reviewer #1: Yes**

**3. Have the authors made all data underlying the findings in their manuscript fully available?**

The PLOS Data policy requires authors to make all data underlying the findings described in their manuscript fully available without restriction, with rare exception (please refer to the Data Availability Statement in the manuscript PDF file). The data should be provided as part of the manuscript or its supporting information, or deposited to a public repository. For example, in addition to summary statistics, the data points behind means, medians and variance measures should be available. If there are restrictions on publicly sharing data—e.g. participant privacy or use of data from a third party—those must be specified.

**Reviewer #1: Yes**

**4. Is the manuscript presented in an intelligible fashion and written in standard English?**

PLOS ONE does not copyedit accepted manuscripts, so the language in submitted articles must be clear, correct, and unambiguous. Any typographical or grammatical errors should be corrected at revision, so please note any specific errors here.

**Reviewer #1: Yes**

**5. Review Comments to the Author**

Please use the space provided to explain your answers to the questions above. You may also include additional comments for the author, including concerns about dual publication, research ethics, or publication ethics. (Please upload your review as an attachment if it exceeds 20,000 characters)

**Reviewer #1:** This is an important contribution and should be published. However, there are suggestions for the authors that would (in my opinion) make the article more useful and interesting.

**Overall Comments:**

**Comment 1:** Key points of uptake of IANC and SBA and their determinants are well made.

**Comment 2**: The overall article can be condensed into a shorter version that is more pithy but also more readable.

**Answer:** Please check the revised manuscript, in which we have tried to reduce contents and make the paper more precise and readable.

**Technical Comments:**

**Question 1**

Given that IANC is an important concept and likely one that the authors have constructed (or used a novel approach), it would be good to describe a bit better how it was determined what IANC is. For e.g., the difference between one or more of the 6 criteria, did they apply some score and a cut off etc. or was it if so and so criteria were met.

**Answer:** It has now been explained with more detail in the paper that the ideal ANC (IANC) was used to reflect the women who received the ANC meeting the following criteria:

ANC that meets all of the following criteria: 1) four or more ANC visits to a skilled healthcare professional during pregnancy, with at least one visit in the first trimester; 2) laboratory tests of blood and urine carried out at least once; 3) blood pressure measure on each visit; 4) prescription of iron tablets; 5) counselling on nutrition during pregnancy; and 6) at least one dose of tetanus toxoid vaccination.

May be useful to think of a comparison between de facto def of ANC vs. IANC (suggestion, not necessarily for this paper)

**Answer**

de facto ANC (Antenatal Care) is the visit of a pregnant woman with a healthcare regardless of the quality of the visit (The previous Pakistan Demographic Health Survey had multiple variables and there was no method to find out the communications skills and medical checkups)

IANC (Ideal Antenatal Care) Since Pakistan Maternal Mortality Survey 2019 was specifically focused on maternal healthcare therefore, the medical checkups and counseling was taken into account thereof the authors labelled it as Ideal ANC as per WHO guidelines to take it as a contact and not simply a visit of patient. It is the visit of a pregnant woman with a skilled healthcare provider that includes the essential medical check-ups and communication to reflect quality.

Would really like an exploration of collinearity between determinants such as education and wealth. Test for collinearity may be reported as part of results section

**Answer**

The collinearity tests in the statistical analysis section has been addressed in the revised document.

**Discussion may be revisited**.

While there is some discussion of the determinants and their international correlation, it would serve to go a bit more in depth about their implications.

Also, authors may wish to comment on the intersection of these determinants.

Plus a discussion between discordance of the determinants of IANC and SBA may be interesting

Answer:

It has been done and relevant points have been added.

**As an editorial point, would suggest moving suggestions towards the end**. Plus some suggestions such as those related to the Sehat Sahulat Program (national health insurance) may be taken in the context of current limitations - funding plus the fact that it is only for inpatients.

**Answer:** All the suggestions have been incorporated in the discussion and recommendations section.

**Rebuttal Letter 2: Reviewer 2**

**1. Is the manuscript technically sound, and do the data support the conclusions?**

The manuscript must describe a technically sound piece of scientific research with data that supports the conclusions. Experiments must have been conducted rigorously, with appropriate controls, replication, and sample sizes. The conclusions must be drawn appropriately based on the data presented.

**Reviewer #2: Partly**

RESPONSE: Further elaboration has been done according to the comments of the reviewers to justify it.

**2. Has the statistical analysis been performed appropriately and rigorously?**

**Reviewer #2: Yes**

**3. Have the authors made all data underlying the findings in their manuscript fully available?**

The PLOS Data policy requires authors to make all data underlying the findings described in their manuscript fully available without restriction, with rare exception (please refer to the Data Availability Statement in the manuscript PDF file). The data should be provided as part of the manuscript or its supporting information, or deposited to a public repository. For example, in addition to summary statistics, the data points behind means, medians and variance measures should be available. If there are restrictions on publicly sharing data—e.g. participant privacy or use of data from a third party—those must be specified.

**Reviewer #2: No**

RESPONSE: We added the minimal underlying data sets as Supporting Information files.

The Pakistan Maternal Mortality Survey 2019 was a national survey conducted along the same lines as Demographic and Health Surveys are carried out. The reports, as well as the raw data, along with all questionnaires, data descriptions, and technical details are also publicly available on the Demographic and Health Survey website. In the paper, the variables constructed for the present analysis are fully explained, while the frequencies of the source variables are available in the final report (also available for download on the DHS website).

National Institute of Population Studies-NIPS. Pakistan Maternal Mortality Survey. Islamabad/Pakistan: NIPS/ICF; 2020.

https://dhsprogram.com/pubs/pdf/PR128/PR128.pdf

**4. Is the manuscript presented in an intelligible fashion and written in standard English?**

PLOS ONE does not copyedit accepted manuscripts, so the language in submitted articles must be clear, correct, and unambiguous. Any typographical or grammatical errors should be corrected at revision, so please note any specific errors here.

**Reviewer #2: No**

RESPONSE: The manuscript has been heavily revised and edited according to the valuable instructions provided by the Reviewers.

**5. Review Comments to the Author**

Please use the space provided to explain your answers to the questions above. You may also include additional comments for the author, including concerns about dual publication, research ethics, or publication ethics. (Please upload your review as an attachment if it exceeds 20,000 characters)

**Reviewer #2:** Overall the paper is structured well. However, what one expects from a secondary analysis paper is the inference from results and a set of recommendations with a way forward.

Neither discussion nor conclusion give any solid recommendations and a way forward.

Limitations are part of Discussion, not the Conclusion.

Conclusion section again discusses the results which is unnecessary. Only last line gives a very generic recommendation which should be unpacked to inform the policy makers and program managers: what is meant by multisectoral approach from health & education sector? Is there any example from Pakistan or region from where a model may be picked up and replicated /adapted.

This is a very important topic so authors must give thread bear details

RESPONSE: We are grateful for pointing out these problems. In the revised manuscript, we have tried to address each of the above remarks as best as possible.
