# Supplementary material for: Factors associated with maternal health services utilization in Pakistan: Evidence from Pakistan maternal mortality survey, 2019
Source: PLoS One. 2023 Nov 16;18(11):e0294225. doi: 10.1371/journal.pone.0294225 (PMC10653445; doi:10.1371/journal.pone.0294225)
Supplement: Attachment — Submitted filename: Response to reviewers.docx [file pone.0294225.s004.docx]

**REVIEW COMMENTS TO THE AUTHOR**

Please use the space provided to explain your answers to the questions above. You may also include additional comments for the author, including concerns about dual publication, research ethics, or publication ethics. (Please upload your review as an attachment if it exceeds 20,000 characters)

**Reviewer #1: The authors have improved upon the original submission by addressing the discussion section. However, some important considerations remain and must be addressed:**

**Reviewer #1: Comment**

1. **They show** that only around a fifth of the women avail IANC while 2/3 avail SBA. Statistically but more importantly contextually that makes these two very different phenomena. For example, age seems not to change SBA and there is no dose-dependance of education or parity suggesting (when there is a dramatic dose effect of over 6 children elsewhere), demand issues. It would useful for the authors to describe what is similar and different, in terms of which women avail which service, location, demographic and the channel of service delivery.

**Answer: 1**

There are 2/3 of women who have availed SBA services, but it does not mean that they had an Ideal ANC (IANC). We have explained that the trends of increasing use of ANC and SBA are parallel. However, the quality of ANC received by women is in question, which we wish to highlight. Ideal ANC means that they have the ANC visits & the required investigation in addition to the utilization of SBA services, the standards as explained below:

- four or more ANC visits to a skilled healthcare professional during pregnancy, with at least one visit in the first trimester;
- laboratory tests of blood and urine carried out at least once;
- blood pressure measure on each visit;
- prescription of iron tablets;
- counselling on nutrition during pregnancy; and
- at least one dose of tetanus toxoid vaccination. SBA was defined as the delivery conducted by a skilled birth attendant including doctor, nurse, midwife, community midwife, or Lady Health Visitor (LHV).

We argue that the quality of ANC is more important than simply visiting the health facility during pregnancy without being properly checked for potential risk factors, receiving iron-folic acid supplementation, getting anti-tetanus vaccination, and being informed about nutrition.

**It would useful for the authors to describe what is similar and different, in terms of which women avail which service, location, demographic and the channel of service delivery.**

The women who use SBA services are mostly affected by wealth quintile and education. The discussion points is that women in Baluchistan, Sindh and GB have relatively lower SBA services because of the longer distances to health facilities although other factors like parity and education are also in play.

**Reviewer #1: Comment**

2. **A recommendation** is made for reaching poor and rural women. However, their results show no difference in utilization by location. Further, given that over half of Pakistan's urban population lives in an urban slum (which would not be identified in this survey, it is likely that their being underserved is not being highlight (given the wealth and education associations that are described). This would suggest that the recommendation should be to focus predominantly on urban poor

**Answer: 2**

We have pointed out that both poverty and residence in rural areas are independent risk factors, and should be addressed. This impact is independent of the province of residence. There is definitely marked difference in rural and urban women.

a) The table 1 shows that urban women utilize IANC (27.9%) and rural women use (15.5) which shows significant difference.
b) Our study suggests strong relationship with lowest wealth quintile (only 3% IANC)
c) Yes, definitely urban poor should be the focus in a province, but since the data of PMMS was not segregated in the urban slums therefore this can be a limitation of the present study.

**Reviewer #1: Comment**

3. **There is some discussion** of supply side interventions. It is unclear given the correlates for low IANC that are described in the regression would mean much more utilization of better trained or equipped providers/ facilities would lead to higher uptake. It seems from the results presented that the problem is a lack of demand and recommendations may address that.

**Answer: 3**

We respectfully disagree and say that this is not the impression coming out of our results. We are focusing on quality of ANC rather than simply the supply of it; hence, it is a supply-side issue. If we will stress on IANC services, that will automatically include SBA services.

Additionally, if the pregnant women reach the health facilities and interact with the health system including a contact with the SBAs, this would increase their likelihood of opting for institutional delivery rather than home delivery by an untrained birth attendant.

**Reviewer #1: Comment**

4. **A similar argument** may be made for SBA. Pakistan saw rapid rise in uptake of SBA between 2012 and 2017, that was attributed by many to supply side interventions such as better equipped and 24/7 facilities. That advantage has been realized. Perhaps a more pronounced discussion of demand creation for both IANC and SBA, given demographic features of low utilizers may be proposed.

**Answer: 4**

Our focus is quality of ANC. However, we have mentioned interventions like Sehat Sahulat Program in the discussion and conclusion.

**Reviewer #1: Comment**

5. **The discussion** is still a bit long, repetitive (both of results and arguments) and circular (argument order need to be revisited for logical flow). It can be cut by at least

**Answer: 5**

We emphasize the importance of giving attention to the association between each factor and ANC.

**Reviewer #1: Comment**

6. **I am not sure** I agree with supply side recommendations that are presented, given the above points. These must be revisited to include a more sophisticated inclusion of context and demand issues

**Answer: 6**

Definitely the supply side recommendation is needed, which we have highlighted that through LHWs and other community-based workers efforts should be made to improve knowledge of women about the importance of ANC services. There should also stress on the importance of blood tests and other necessary services.

**Reviewer #2: Authors have made revisions that are acceptable and changes made are fine for the manuscript to be accepted.**
