# Supplementary material for: Factors associated with maternal health services utilization in Pakistan: Evidence from Pakistan maternal mortality survey, 2019
Source: PLoS One. 2023 Nov 16;18(11):e0294225. doi: 10.1371/journal.pone.0294225 (PMC10653445; doi:10.1371/journal.pone.0294225)
Supplement: Attachment — Submitted filename: Response to reviewers.docx [file pone.0294225.s006.docx]

**REVIEW COMMENTS TO THE AUTHOR**

Reviewer #3: Overall the study addresses a critical issue related to maternal health in Pakistan and offers valuable insights into the utilization of ANC and SBA services. The research design is sound, but would benefit from additional details.

The introductory section exhibits a conflation of themes pertaining to LMICS (Low- and Middle-Income Countries) and Pakistan. In light of this, a more suitable approach would entail a restructuring of the introduction, wherein the discussion initially centers on LMICS and subsequently narrows its focus to Pakistan. Given that the manuscript emphasizes the importance of quality ANC, it would be advantageous for the introduction to be more specifically focused on this aspect of ANC and SBA. However, it lacks a clear statement of the study's objectives and hypotheses and how the research contributed to the already established literature on the topic. This could be beneficial to guide readers through the research focus.

The methods section provides an adequate description of the data source and study variables. However, the section should elaborate on the rationale to support the use IANC. The services described in the constitution of IANC does not encompass the broader concept of quality and patient safety. Therefore, it is imperative that the authors explicitly specify the usage of "quality" and "ideal" ANC. It is suggested to provide a sample frame selection framework and number of participants for the two outcomes. It would be beneficial if the univariate and bivariate variables selection criteria for the multivariate model can be stated in the methods sections.

The results section is well documented. However, it is suggested to present descriptive statistics as number and %.

The discussion provides a comprehensive analysis of the study's findings and relates them to existing literature, offering a valuable contribution to the field. However, it needs minor revision to curtail the use of overwhelming statistics. To improve the clarity and readability of the section, the authors should consider organizing the statistics into concise and well-structured sentences. This approach will help the readers better grasp the key findings without getting lost in a sea of numbers. Discussion could be further strengthened by including a critical evaluation of the possible sources of bias. Additionally, the discussion would benefit from a more explicit connection between the study's results and the policy implications proposed.

**Reply:**

The introduction and discussion sections have undergone revisions, along with the addition of a few references to support the introduction and explanation of definition for ideal ANC.

Reviewer #4: Editing, copywriting and proofing from a Native English language editor is recommended for the paper, for overall language of the manuscript is weak and needs improvement.

**Reply:**

The language has been refined. I trust it has been improved.
